# Trajectory of smoking behavior change among Chinese immigrant smokers

**DOI:** 10.1371/journal.pone.0246280

**Published:** 2021-02-02

**Authors:** Fang Lei, Eunice Lee, Ying Zheng

**Affiliations:** 1 School of Nursing, University of California at Los Angeles, Los Angeles, California, United States of America; 2 Department of Health Care Quality Improvement, Shenzhen Nanshan Medical Group Headquarter, Shenzhen, China; Faculty of Health Sciences - Universidade da Beira Interior, PORTUGAL

## Abstract

**Background:**

The incidence and mortality rates of smoking-associated lung cancer are high among Chinese immigrant smokers. Coming from a country with different smoking policies, culture, and economic background than the U.S., Chinese smokers may change their smoking behaviors after immigrating to the U.S.

**Objectives:**

This study aims to explore the trajectory of smoking behavior change among Chinese immigrant smokers migrating to the U.S.

**Methods:**

This qualitative study was guided by the Grounded theory. Semi-structured intensive individual interviews were conducted among 10 eligible participants. A purposive theoretical sampling method was used to recruit participants through a website. Individual interviews were conducted online, via telephone, or in-person in Mandarin. Data were transcribed verbatim in Mandarin, translated into English, and triangulated with follow-up interviews and dialogues among authors to enhance trustworthiness and consistency of the study. Process coding and content analysis were used to analyze data.

**Results:**

A total of 10 participants, 7 males and 3 females, were interviewed. Results showed the trajectory of smoking behavior change among Chinese immigrant smokers went through three phases: 1) Beginning to smoke, which included learning to smoke from others, trying to smoke and learning to smoke, and hiding their smoking behaviors from others; 2) maintaining smoking, which included setting boundary around smoking, smoking as a facilitator or barrier to social interaction, feeling pressures related to smoking behavior, and making others happy: Collective smoking and controlling smoking desire; and 3) changing smoking behaviors, which included experiencing life events that were triggers to changing smoking behavior, boredom as a reason for relapses, personal will as a key to quitting smoking, and quitting smoking for a loved one. Although some differences existed between male and female Chinese immigrant smokers’ smoking behaviors, their trajectories of smoking behavior change were generally similar.

**Discussion:**

Findings from this study can help health care providers to extend their understanding toward smoking behavior change among Chinese immigrant smokers across different socio-cultural contexts.

## Introduction

Lung cancer is the second most commonly diagnosed cancer in both males and females in the United States (U.S.) [1]. It is also the leading cause of cancer deaths in the U.S. population [1] and among Chinese Americans [2]. In 2011, 14% of cancer patients were diagnosed with lung cancer, and 27% of cancer deaths were attributed to lung cancer [3]. In 2016, 156,176 people died from lung cancer [4]. The mortality rates for lung cancer were 31% and 26% in men and women, respectively, exceeding the mortality rates caused of prostate cancer (10%) and breast cancer (15%) [4]. As the second and fourth most common cancer among U.S. Chinese men and women, respectively, lung cancer accounted for approximately 30% of all cancer-related deaths in Chinese Americans [2].

Cigarette smoking is a major cause of lung cancer. Up to 60 known carcinogens have been identified in cigarette smoke [5]. It contributes to 87% of all lung cancer related deaths among adults in the U.S., with a death rate up to 90% in males and 65% in females [6]. The duration of smoking, the number of cigarettes smoked, and exposure to secondhand smoke are positively associated with the risk of lung cancer [5]. The number of adults who are aged 55 to 75 years in the U.S. with a smoking history of 30 pack-years or more is about 8.6 million [7]. Although data about the smoking rates among Chinese immigrants were limited, smoking remains a major public health issue that is particularly severe among Chinese Americans. Smoking causes high mortality among Chinese Americans [8]. Compared to the smoking rate in general population of the U.S. (13.7%) [9], the smoking rate among Chinese American population is high, ranging from 17.36% [10] to 17.98% [11]. The smoking rate was much higher in Chinese American men (29–34%) than in Chinese American women (2–4%) [10,11], whereas the smoking rate was 15.6% for males in the U.S. and 12.0% for females [12].

Smoking cessation is the most efficient way to decrease lung cancer incidence and mortality rates [7,13]. In 1995, smoking was initially banned in all enclosed workplaces in California. In 2012, smoking restrictions in rental residential dwellings was passed and written into California law [14]. As of July 2018, 26 states had enacted statewide bans on smoking in all enclosed workplaces, including all bars and restaurants [15]. In China, the Ministry of Health of China completely banned smoking in all health administration offices and medical facilities in 2011 [16]. However, no policy to ban smoking in residential areas has been implemented in China. As the social environment after immigration changes significantly and the policies related to smoking regulations differ between the U.S. and China, Chinese immigrants may change their smoking behaviors after immigration [17]. However, until now, little was known about Chinese immigrant smokers’ smoking behavior after immigration [18].

Although factors influencing Chinese immigrant smokers’ smoking behavior are unclear, previous research identified various factors that influenced Chinese Americans’ smoking behavior, including language proficiency [19], education level [11], social smoking norms [20], depression [21], acculturation [22], knowledge about smoking consequences [23], and perceived benefits of quitting smoking [24]. Among 167 Chinese adult immigrant smokers in Vancouver, Canada, a quantitative study showed that education level, support from health care providers, and access to smoking cessation programs had an impact on their perceptions of quitting smoking [24]. Among Chinese-speaking smokers in California, a qualitative interview study identified that irritating odor, smoking cessation encouragement from physicians, benefits of cessation on family harmony and children’s health, beliefs about harms of smoking, acculturation and recognized changes in environmental regulation and social acceptability of smoking after immigration impacted intentions of smoking cessation [25].

Although these studies added important evidence for Chinese immigrant smokers’ perceptions of smoking cessation, whether they changed their smoking behaviors after immigrating to the U.S. and what impacted Chinese immigrant smokers to change their smoking behaviors were not clear. Considerable variation in experiences of smoking behavior change may exist between Chinese immigrant smokers and other ethnic groups. Understanding Chinese immigrant smokers’ trajectory of smoking behavior changes could help us identify factors influencing their smoking behaviors and the development of effective strategies to decrease the smoking rate among this population. Also, targeted smoking cessation interventions by country of origin [26] can make important contributions to public health and toward building a healthy environment [27].

The purpose of this study is to investigate Chinese American smokers’ trajectory of increases or decreases in cigarette smoking. The research question for this study was: What is the trajectory of Chinese immigrant smokers’ smoking behavior change, before and after they immigrated to the U.S.?

## Materials & methods

### Design

This qualitative study used a semi-structured intensive individual interview method. The grounded theory was used to guide the individual interview and data analysis processes.

### Protection of participants’ rights

The Institutional Review Board (IRB) at University of California Los Angeles approved this study. The approval number is IRB#17–001124. Written informed consent was obtained from the participants. Materials including the semi-structured interview guide, demographic information questionnaire, recruitment flyers, screening script, and consent forms were also approved by the IRB. Participants gave informed consent before the interviews to affirm that they understood they could refuse to answer any question and withdraw from the study at any time. Each participant was assigned a study number. All of the paper documents with participants’ identifiers were locked in a cabinet. Only the research team was able to access the data.

### Theoretical guidance

Procedures of data collection and analysis of this study were guided by the Grounded Theory. The Grounded Theory was developed by Barney Glaser and Anselm Strauss [28]. Being different from the hypothesis-deductive approach, Grounded Theory is an inductive research method [29]. Studies using Grounded Theory often aim to develop a theory grounded in data, which often begins with questions, or even just with the collection of qualitative data [29].

### Sample

Eligibility criteria for the participants were: Chinese immigrants (a person having origins in China) [30] who immigrated to the U.S. within the last 10 years (to avoid recall bias); English or Mandarin speaking; current smoker or ex-smoker; and 21 years old or older. Exclusion criterion for the participants was having a current or past lung cancer diagnosis.

The sample size of this study was determined by data saturation toward the research question. According to Glaser and Strauss [28], the determinative procedure of sample size is quite different for qualitative study than quantitative study. Reaching the “theoretical saturation” is the determinant for sample size [28]. Although the quantity of available descriptive data from each participant is disparate and the quality of data is various [31], after interviewing 10 participants individually, data for this study reached “theoretical saturation”.

### Recruitment

The participants were recruited from one of the most frequently visited websites (www.chineseinla.com) among Chinese Americans. On the website, there were multiple forums for Chinese Americans to share their life experiences. Within the “life in a foreign country,” IRB-approved informational flyers describing the purpose of study, inclusion/exclusion criteria, reimbursement amount for interview time, and the primary investigator’s contact information (email and phone number) were posted both in English and Chinese.

When a Chinese American who was interested in participating in the study called the primary investigator’s telephone number listed in the flyer, s/he was screened for eligibility by the primary investigator. Screening questions related to age, smoking history, immigrant status, original country, language, and medical history were asked to identify eligible participants. Participants’ telephone number and email address were collected as needed. Eligible participants had the option to be interviewed online, via telephone, or in person, depending on their preferences. If the participant chose to be interviewed online or via telephone, they received an informed consent form online. The demographic information questionnaire was sent to participants after we received the electronic version of their signed inform consent form. If the participant chose to be interviewed in person, they were required to read and sign the informed consent form and fill in the demographic information questionnaire before the interview.

### Procedures

A semi-structured interview guide was used to guide the interviews (Table 1). The interviews were conducted by the primary investigator, who is a bilingual and a bicultural nurse researcher. The interviews were conducted in person, online, or through phone, depending on the participants’ preference. In-person interviews were conducted in a quiet place in the primary investigator’s community. During the interview, open-ended prob questions were asked without imposing any opinions from the researcher which may potentially limit the inquiry. The interviews were recorded on a digital recorder or through phone, and appropriately labeled without participants’ identifiers. The documents were downloaded into a laptop secured by password for coding and analysis. A number was assigned to each transcript to protect participants’ personal information from accidental disclosure. All of the interviews were conducted in Mandarin. Data were transcribed verbatim and translated to English by two bilingual, bicultural researchers. The primary investigator and another author verified the accuracy of the transcriptions. After the interview, $25 was given to the participant in person or online to compensate their time and effort.

**Table 1:** Semi-structured interview guide.

1. Could you please tell me your story about the reasons why you start smoking?
2. Please talk about your experience when you smoked most of the cigarettes before you immigrated to the United States. What was the occasion you often smoked?
3. Please talk about your experience when you smoked most of the cigarettes after you immigrated to the United States. What was the occasion you often smoked?
4. Tell me about the first time you realize that you have to change your smoking behavior. What impact do you think it has on your life after you change your smoking behavior?
5. Take a moment to think of some of the things that most affected your smoking habits. What has affected it the most?
6. Some people think smoking can help them relax from stress, but some others think it is not good for health, how about your opinion on smoking?
7. For some people quitting is hard and for others it is easy. Some people begin smoking again soon after quitting and others do not. Some people quit many times or continue to smoke on and off again. Please tell me about your experience with quitting smoking. What has that been like for you?
8. Some people tried going to see a doctor, using the smoking cessation health service (e.g., smokers’ helpline), and smoking cessation assistant methods (e.g., e-cigarettes) to quit smoking. How was that in your case?
9. How much information have you got on smoking cessation from your doctors, nurses or community health workers?
10. How much information have you got on smoking cessation from your family members? For example, when your family members saw you smoking, what did they say?
11. What are the barriers for you to quit smoking?
12. Is there anything else you want me to know about your experience and perceptions of smoking behavior change?

In the data collection and analysis processes, the primary investigator’s observations and feelings through the interviews were recorded in field notes and memos. The field notes and memos for each participant were taken before, during, and after the interviews through each interaction (including telephone calls, informed consent signing, interviews, and follow up interactions). The participants’ study numbers instead of identifiers were included in the field notes. Insight gained from data analysis was recorded to help to explore the topic further by adding, subtracting, or modifying interview topics and questions.

### Trustworthiness and consistency

To guarantee the rigor of this study, research questions were specifically designed and revised by referring to the advice from peers and experts with a similar research area. Digital records and memos were taken during the data collection and analysis procedures. Digital interview contents were transcribed, translated and verified by two researchers separately. Situational maps including a messy map and an ordered map as well as diagrams were drawn to help the data analysis process.

### Data analysis

The data analysis procedures for this study were guided by Grounded Theory. Four coding phases (initial, focused, axial, and theoretical coding) were conducted in the data analysis processes. Initial coding was done through line-by-line coding of the data in each interview [32]. Focused coding was done to compare data with data and identify the most frequent and significant codes. Axial coding was used to link the subcategories, properties, and dimensions within each category. Theoretical coding was done at the final phase to identify possible relationships between categories [32]. In this study, process coding and content analysis were specifically used to analyze data in each coding phase. First, the transcripts were manually coded by a bilingual, bicultural researcher and the primary investigator using line-by-line initial coding. Then, focused coding was conducted across the transcripts. The events related to the focused coding in each interview were colored using different colors and classified into similar categories. Under the same category, similar properties and dimensions were categorized together. New themes and categories were added to the existing themes and categories until no themes or categories emerged from the data.

## Results

### Sample characteristics

Twelve participants called the primary investigator and expressed their interests in participating in this study. However, two participants were not eligible for the study because of their non-smoking history. Ten interviews (7 male interviewees and 3 female interviewees) were completed, with a mean length of 55 minutes each (range: 45–80 minutes). Mean age of the participants was 43.2 years (range: 39–51 years). Among the 10 participants, 50% were current smokers, 70% had a less than 30 pack-year smoking history, and 80% smoked fewer than 10 years. All of the participants smoked less or quit smoking after they moved to the U.S. More demographic characteristics can be found in Table 2.

**Table 2:** Sample characteristics.

Item	Category	N (%)
Age (y)	39–51	Mean = 43.2
Gender	Male	7 (70)
	Female	3 (30)
Marital status	Single	4 (40)
	Married	5 (50)
	Divorced	1 (10)
Number of children	0 child	5 (50)
	1 child	4 (40)
	2 children	1 (10)
Educational attainment	High school graduate	1 (10)
	Some college	3 (30)
	College graduate	6 (60)
Annual income	$10,001 - $30,000	8 (80)
	$30,000 - $50,000	2 (20)
Insurance	Medical or Medicare	5 (50)
	Private insurance	2 (20)
	No insurance	2 (20)
	Company insurance	1 (10)
Residence years (y)	1–3	4 (40)
	4–6	4 (40)
	7–10	2 (20)
English-language proficiency	Can't speak	5 (50)
	Basic speak	5 (50)
	Can't listen	3 (30)
	Basic listen	7 (70)
	Can't read	3 (30)
	Basic read	7 (70)
Smoking history	Smoke everyday	2 (20)
	Smoke occasionally	3 (30)
	Quit smoking in 15 years	5 (50)
Smoking status	Current smoker	5 (50)
	Former smoker	5 (50)
Smoking length (y)	Less than 1	1 (20)
	4–6	4 (50)
	7–10	3 (30)
	More than 10	2 (20)
Cigarettes package index	Fewer than 30 package years	7 (70)
	More than 30 package years	3 (30)

### Qualitative results

**Fig 1:**
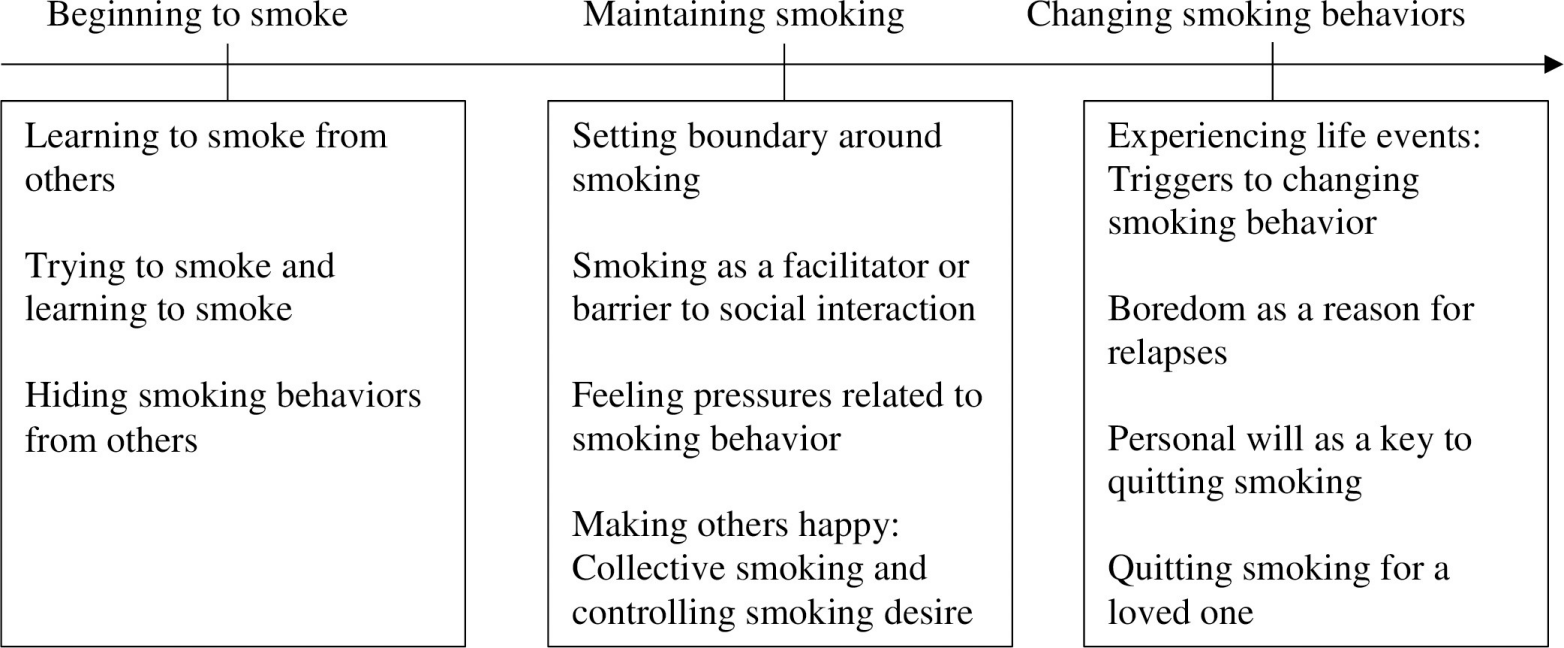
Trajectory of smoking behavior change among Chinese immigrant smokers.

The trajectory of smoking behavior change among Chinese immigrant smokers was categorized into three phases: Beginning to smoke, maintaining smoking, and changing smoking behaviors (Fig 1). Results showed that all of the participants began to smoke in their adolescence in China. They maintained their smoking behaviors for a certain period of time and changed their smoking behavior after experiencing certain life events such as immigration, getting a job, etc.

#### Beginning to smoke

*Learning to smoke from others*. Most male participants started smoking when they were 16 to 18 years old. They learned smoking from their classmates, friends, parents, or movie stars. Their learned smoking behavior may be related to the stress or adolescent rebelliousness. Some participants described stress from study and college entrance examination as a reason they started smoking. Other participants learned to smoke to have fun and play. It seemed that their curiosity about smoking motivated them to smoke in the initial phase. However, female participants tended to smoke at a later age (over 20 years old) after they began to work. The main reason reported by them was simply life stress. Even though difference existed between female and male participants’ initial smoking behaviors, all of the participants learned to smoke by imitating others’ smoking behaviors.

*Trying to smoke and learning to smoke*. When talking about their process of beginning smoking, all participants described the process as “tried to smoke and learned how to smoke.” Although they were afraid of punishment from teachers and parents, they eventually tried and learned to smoke at the same time. Some male participants tried to smoke by taking their friends’ suggestion that “it is OK to smoke, just try.” Other male participants thought smoking was a normal behavior among boys and men. They tried to smoke to show their adulthood, flaunt their superiority. Female participants, while they were aware that their smoking was not generally accepted, still tried to smoke several times until they got used to smoking. Although all of the participants thought “the feeling of smoking is not good” when they first tried to smoke, they still tried to smoke several times, learned to smoke, and kept on smoking.

*Hiding smoking behaviors from others*. Most participants described their smoking behavior during the learning phase as “sneakingly.” Some male participants smoked in the restroom during the class break, so their parents and teachers would not know. Most of the female smokers participated in this study concealed their smoking behaviors from their acquaintances all the times (both in China and in the U.S.) until they quit. They smoked without their parents, husband, or children knowing until they quit smoking. One female participant described her concealed smoking behavior as not wanting to cause unnecessary trouble because “Chinese people have some negative perspectives on females’ smoking behavior”.

#### Maintaining smoking

*Setting boundary around smoking*. All of the participants had a clear boundary around their smoking behaviors when they were interviewed. The participants who had children never smoked at home. Some participants never smoked in friends’ homes. Other participants who got smoking cessation suggestions from their health care providers never smoked during the treatments. Before immigration, the participants had their personal boundary around smoking (e.g., not smoking in the presence of children or in friends’ homes). After immigration, all of the participants perceived the public conventional smoking boundaries. They followed the smoke-free policy and “never smoked in the public areas.” They controlled their smoking craving when they realized the existence of prohibitions.

*Smoking as a facilitator or barrier to social interaction*. All of the male participants in this study described smoking as an efficient way to communicate with others before they immigrated to the U.S. They smoked when they had social interactions with other people. They believed smoking was a facilitator to their social interaction with other people in China. One participant said,

‘Cigarettes is the communication way between men…’ ‘Come on, smoke a cigarette.’ When we ignite cigarettes, we have more things to talk. ‘No, I do not smoke.’ Then others will not talk to you. That is to say, cigarettes are the communication tool between men. Such as, ‘Borrow a cigarette, ignite the cigarette.’ Then the two persons will talk to each other. If you say, ‘I don’t have a lighter. I do not smoke.’ You do not smoke, then the others will not talk to you. They will talk to others.

Conversely, the female participants in this study thought smoking was a barrier to their social interactions with others in China. To conceal their smoking behaviors, female participants reduced their social interaction with others (e.g., colleagues). One of the female participants kept away from her colleagues as far as possible. She did not eat with her colleagues for years. So, they all thought she was an unsocial person.” Smoking “impacted her interpersonal relationship” and “was not helpful” in her social interactions with others.

After migrating to the U.S., the male participants perceived fewer benefits from smoking in their social interactions with others. Some male participants even perceived stigma around smoking in public, which turned out to be a barrier toward their social interaction with others. For the female participants, after migrating to the U.S., they perceived fewer barriers to smoke in their social interactions with others. One of the female participants said that after she migrated to the U.S., she “smoked when she went out for a walk around [her] community. It had no impact on [her] social interaction with others”.

*Feeling pressures related to smoking behavior*. All of the participants in this study learned to smoke before they migrated to the U.S. Some male participants felt peer pressure to smoke which led them to start when they lived in China. After they migrated to the U.S., the participants began to become aware of the smoke-free policy. They acknowledged the restriction against smoking in public areas, which caused them to control their craving and facilitated smoking cessation.

For female participants, after they migrated to the U.S., they felt much less stressed when they smoked. As one of the female participants said, even though she was “still worried that her husband may discover her smoking behavior, she felt less pressure from the society and outside environment”.

*Making others happy*: *Collective smoking and controlling smoking desire*. Although all of the participants thought smoking cessation was an individual issue, they thought smoking was not just their own personal issue both before and after immigration. When they smoked, they nearly always considered people’s feelings. Some participants smoked to “join their friends.” They thought it was not good to refuse others. Some other participants controlled their smoking behaviors to avoid annoying other people when they could not find an appropriate time and occasion to smoke. One participant said, “It (smoking) has no effect [on my life] at all. But it makes others happy, which determined whether I smoke or not at that time”.

#### Changing smoking behaviors

*Experiencing life events*: *Triggers to changing smoking behavior*. In this study, all of the participants’ smoking behavior change was related to events that happened during their lifetime. Overall, all the participants smoked less or quit smoking after they moved to the U.S. Some participants changed their smoking behavior after they went to college in China. They tended to smoke individually instead of smoking collectively. At times, some participants tended to smoke more cigarettes when they felt pressure or stress from their jobs (in China), while other participants smoked less due to their bosses’ dislike of smoking (in the U.S.), etc. Some participants changed their smoking behaviors to adjust their relationships and personal roles in their family (e.g., becoming a parent). One of the female participants quit smoking after she became pregnant. Another participant smoked less after he met his girlfriend.

*Boredom as a reason for relapses*. Most of the participants (80%) in this study had the experience of relapse. Among the 10 participants in this study, 6 participants described life in the U.S. as boring. Some participants felt they had nothing else to do in their spare time. One participant said “smoking is nothing. It is a hobby.” Another participant said, “Boredom. When I have some work to do, I will not think about smoking. Just boring. When I have spare time, then, I want to smoke”.

*Personal will as a key to quitting smoking*. All of the participants in this study thought personal will was the most important factor for smoking cessation. Some participants only relied on their internal willpower to quit smoking. They were not willing to seek any external help or use any smoking cessation assistance. Some participants tried the nicotine patch but later they stopped using it and decided to quit smoking on their own willpower. Some participants thought quitting smoking is an individual issue that could not be forced upon them or helped by others. They tended to quit smoking on their own.

*Quitting smoking for a loved one*. In addition to personal will, some participants mentioned the importance of the motivation in quitting smoking. They thought the key is quitting smoking for a loved one. Some participants quit smoking for their children. One participant quit smoking for his girlfriend. One participant said, “Quitting smoking for your loved one. Although I am single now and I do not have a girlfriend, I do not want to be in this condition for my whole life”.

## Discussion

To our knowledge, to date, this is the first study exploring the trajectory of Chinese immigrant smokers’ smoking behavior change across China and the U.S. Results showed that at the initial stage of smoking behavior change, most of the male participants’ motivation for learning to smoke derived from their adolescent rebelliousness and curiosity. Although the rules in their schools and families both prohibited them from smoking, their smoking behaviors were still influenced by external human and nonhuman factors. Although they didn’t feel well with the cigarette smoke, they still thought it was impolite to refuse to smoke when somebody offered them cigarettes. They tried and learned to smoke, but they were afraid of the punishment from teachers and parents at the same time. These paradoxical mental activities reflected the possible confusion around smoking in adolescence. Developing tailored education programs for adolescents may function as an essential way to raise their awareness of staying away from smoking. For example, utilizing multiple education methods (e.g., cartoons, visual images) and materials with appropriate health literacy level could increase adolescents’ knowledge of the consequences of smoking. Furthermore, smoking should be perceived as an unhealthy and a bad habit instead of a “cool” habit among adolescents. Targeted intervention programs could help adolescent smokers to quit smoking at the early stage.

Chinese immigrant smokers’ maintaining smoking behaviors may be associated with the etiquette in China. Results showed that the prevalence of smoking among a specific gender (male or female) or group (adult or adolescent) was closely associated with cultural acceptance of smoking by the public. While smoking behaviors among adolescent and females were not well accepted in Chinese culture, smoking among adult males was widely accepted [33,34]. As a country with a long history and culture related to cigarettes, China has a great tolerance for cigarette smoking among males [33,34]. As the participants said, smoking was not just an individual behavior, but a collective behavior. It functioned as a communication method among adult males. It provided an opportunity for the adult male smoker converse with others, facilitating social interactions and relationships. However, for the adolescent and female smokers, the opposite was true. Smoking among adolescents and females was not accepted by the public [33,34]. Female smokers tended to hide their smoking behavior, which greatly impeded their social interactions with others. Eventually, their self-isolation and hidden smoking may increase their stress level and the possibility of addiction, which may lead to a vicious cycle of smoking, social isolation, and stress.

In addition, Chinese immigrant smokers’ smoking behavior seems driven by their moral priorities. Being born in a country that viewed etiquette as the primary interpersonal communication principle, Chinese immigrant smokers valued their relationships with others [35], which may influence the direction of their smoking behavior change. As the data show, the participants valued their family members’ health, especially children’s health. They had strict boundaries around their smoking, controlling their nicotine cravings when they visited their friends’ homes. They chose not to smoke when the place and time were not appropriate. They were also motivated to quit smoking for loved ones. For them, making others happy is the moral priority. It was more important than their craving of cigarettes. This moral priority driven smoking behavior change was consistent with the previous study [35], which identified moral priorities as a factor impacting Chinese’ smoking behavior.

Furthermore, smoking relapse seems prevalent among Chinese immigrant smokers. Contrasted to the stress and craving for cigarettes, which were usually mentioned as the reasons for smoking relapses among the U.S. population [36], participants in this study attributed smoking relapses to boredom. This is consistent with the findings in other studies focusing on the Chinese population [37,38]. Notably, even though the smoking relapse rate (80%) was high among this population, the utilization of smoking cessation assistance (nicotine patch, smoking cessation hotline) was not sufficient. Instead of using external help, participants in this study relied on their willpower to quit smoking.

### Limitation

Like most qualitative studies, this study has some limitations. First, as the participants’ previous experiences were described retrospectively through semi-structured interviews, recall bias may exist. Second, with the smoking behaviors decreasingly accepted by the public, the participants may have exaggerated some details to ingratiate him- or herself with the researcher, bring self-reported bias to the data. Lastly, the education level, residence year, income, and English proficiency may influence Chinese immigrant smokers’ smoking behavior, but given the data were analyzed by Grounded Theory, triangulation of the quantitative data and qualitative data is not feasible in this study.

### Future directions

Although this study had several limitations, it informs future direction for research and clinical practice. First, findings from this study suggest that culturally targeted intervention programs need to be implemented for Chinese immigrant smokers. Culturally tailored smoking cessation assistance education programs which are developed in Chinese language and formulated with an appropriate health literacy level are needed. Also, reframing etiquettes associated with the ‘cigarette culture’ and emphasizing the importance of significant others in smoking cessation process are essential to facilitate smoking cessation. In addition, during smoking cessation consultation, Chinese immigrant smokers’ moral priorities need to be taken account to by health care providers. Individually tailored suggestions with considerations of their contextual situations (e.g., consideration of children’s health) and advices on the enrichment of mental health status to overcome the boredom (e.g., connection with the community activities resources) should be offered to help them to quit smoking or reduce the likelihood of relapse. Second, special intervention programs need to be developed to help Chinese female smokers to overcome the barriers to social interaction brought on by smoking. Ending the vicious cycle could be the key to reduce smoking rate among this population. Tailored but highly confidential smoking cessation programs utilizing smoking cessation consultation, yoga, meditation and other exercise projects to decrease stress but without naming it obviously as the smoking cessation programs should be implemented to help the female smokers to decrease stress and improve social interactions with peers. Lastly, future research focusing on the exploration of factors that statistically impact immigrant smokers’ smoking behaviors, including moral priorities (e.g., making others happy) for Chinese immigrant smokers, need to be done to further assist them to quit smoking.

## Conclusions

This study extended the understanding of the trajectory of smoking behavior change among Chinese immigrant smokers across different socio-cultural contexts of China and the U.S. It provided in-depth evidence that may help to develop gender-specific health promotion intervention programs to improve smoking cessation among male and female Chinese Americans. Findings from this study can help health care providers to better understand the determinants that influence Chinese immigrant smokers’ smoking cessation decision, thus facilitating Chinese immigrant smokers’ decision to quit smoking. It can also help health policy makers to design appropriate smoking control polices to decrease the rates of smoking and lung cancer among Chinese Americans.

## Supporting information

S1 File (DOCX)
