# Supplementary material for: Trajectory of smoking behavior change among Chinese immigrant smokers
Source: PLoS One. 2021 Feb 2;16(2):e0246280. doi: 10.1371/journal.pone.0246280 (PMC7853480; doi:10.1371/journal.pone.0246280)
Supplement: S1 File — (DOCX) [file pone.0246280.s001.docx]

**Transcript**

**Interviewer:** Fang Lei

**Interviewee:** Andrew Zen (Pseudo-name)

**Date of Interview:** March 10, 2018

**Start Time of Interview:** 3 pm

**End Time of Interview:** 3:45 pm

**Location of Interview:** UCLA, FH

**Interview Topic:** Experiences and perceptions of smoking behavior

change among Chinese immigrants

Fang: Hello, Andrew. How are you? I am Alice. Thank you for your participating in my research study. Before we start, I would like to introduce my research topic a little bit, so you can know more about what we are doing and the significance of this research study. The research that I am doing now aims to investigate Chinese immigrant smokers’ experience and perceptions of smoking behavior change and lung cancer screening. The change of the smoking behavior refers to the possibility of the increase of the smoking amount, or the possibility of the decrease of the smoking amount. This study will provide valuable data for developing tailored education and intervention programs in the future. Your participation today will help more people at high risk to prevent lung cancer in the future projects. In our interview, I will use two recorders to record our conversation. The purpose of recording is to transcribe and analyze data. Your talking content is confidential. No personal information can identify you in my research and publication. This interview will last less than one hour. If any question during the interview makes you feel uncomfortable, you can choose not to answer. Also, there is no right or wrong answer regarding each question. OK, are you agree to use recorders to record our conversation?

Agreeing to use recorder to record the conversation

Having no question about the research

Having no question about the informed consent

Agreeing to participate in the study

Agreeing to be contacted for further research programs

Impacting by the media (heroes from movies); Regarding smoking as cool; Hoping to be a hero

Andrew: Yes.

Fang: Is there any question about the research before we start?

Andrew: No.

Fang: As you saw the informed consent just now, do you have any questions on that?

Andrew: No.

Fang: Do you agree to participate in this study?

Andrew: Yes.

Fang: If we have some further relevant research programs that need you to participate, can we contact you?

Andrew: No problem.

Fang: OK. First, could you please tell me your story why you started smoking?

Andrew: In the beginning, I saw some people in the movies, for example, Yunfa Chou. They smoked. I think it is cool. I want to be a person like them. In addition, I think the people who smoke seem quite knowledgeable. So, I want to be the person of their styles. These are the main reasons why I want to smoke.

Linking smoking with knowledgeable; Hoping to be a knowledgeable person

Starting smoking around his 30s

Getting information on smoking from his friends; Friends regarding smoking as fine; Friends recommending him to try to smoke; Getting cigarettes from friends; Not caring about the brands of the cigarettes; Friends not sticking to smoke one brand of cigarettes; Having experience of smoking different kinds of cigarettes

Getting cigarettes from friends; Having no craving for cigarettes

Emphasizing having no craving for cigarettes; Smoking as a way to join his friends; Having no desire to smoke when he is way from his friends

Fang: When was that time when you started to smoke?

Andrew: That time... that was a long time ago. Approximately... let me think. I am 43 years old now. I was probably 29 or 30 years old at that time.

Fang: So, you learned the smoking behavior from media. How about the impact from the people around you? Where did you get the cigarettes? How did you begin to smoke?

Andrew: Some of my friends told me it was OK to smoke, just try. I did not care about the brands of the cigarettes quite much. They gave me one package of the cigarettes occasionally. They smoked different brands of the cigarettes.

Fang: Where did you get cigarettes then?
Andrew: I got cigarettes offered by my friends. I did not have craving for the cigarettes. Some of my best friends bought two to three packages of cigarettes and they gave me one package. Or sometimes they had already smoked several cigarettes in one package, they gave the left cigarettes to me.

Fang: So, you did not buy cigarettes for yourself?

Andrew: I have no craving for the cigarettes, sometimes, in order to join them, I smoked. But if I did not stay with them under that situation, I have no desire to smoke.

Fang: So, you smoked the cigarettes offered by your friends and smoked mainly with your friends. Under other conditions, you did not think about smoking, right?

Andrew: Yes. Because the friends offered me the cigarettes, it is not good to refuse them. In order to keep the same with them, I smoked passively, making myself look the same as them.

Friends offering him cigarettes; Unwilling to refuse his friends; Hoping to be the same as his friends; Feeling social pressures from the interaction with his friends

Locating the occasion when he smoked most before immigration (with friends, at Karaoke, at night); Having boundary on his smoking behavior (rarely smoked in daytime); Wine and cigarettes engaging in the social interaction; Smoking offered cigarettes

Smoking on weekends; Smoking with friends; Being invited to the social interaction occasion; Unwilling to refuse friends (personality or culture?)

Getting away from friends; Living with parents; Smoking less; Locating the occasions he smoked most after immigration (with colleagues, playing table tennis, on weekend); Smoking offered cigarettes; Smoking less cigarettes

Fang: Can you talk about your experience when you smoked most of the cigarettes before you immigrated to the United States? What was the occasion you often smoked?

Andrew: That time... sometimes My friends went to the Karaoke. They sang some songs. Usually, we smoked on that occasion at night. We rarely smoked in daytime. They sang the songs, chatted with each other, drank wine and smoked cigarettes. They gave me the cigarettes and I smoked.

Fang: How many cigarettes did you smoke per day before?

Andrew: Usually, they invited me, for example, during the week, on Friday, Saturday, two days per week. Although they may not go there exactly on Friday or Saturday, if they invited me to go and gave me the cigarettes, I did not refuse.

Fang: OK. Can you talk about your experience when you smoked most of the cigarettes after you immigrated to the United States? What was the occasion you often smoked?

Andrew: After I immigrated ... Because most of my friends live in Taiwan, while my parents and I live here, I smoked less. Very little. I remembered one time. I was working at the factory at that time. They invited me to play table tennis on weekend. They gave me the cigarettes and I smoked. But the number of cigarettes I smoked could not be compared with that when I was in Taiwan, as most of my friends are in Taiwan.

Fang: Tell me about the first time you realize that you have changed your smoking behavior. For example, you said after you came to the United States, you decreased your smoking amount, what did you think when you realized this situation?

Andrew: In fact, when I was in Taiwan, I smoked mainly by considering the relationship with my friends. After I came here, I had not so many friends here. So, it means that I can do my own things. Although I want to be the person in the movies, Gold of Gamblers, very cool. I want to learn to be that people. But there is a problem when I smoke... For example, when I was young, I saw people drinking. They looked cool. But when I drank, the wine was quite bitter. The taste was not good. After I tried several times, I did not want to try anymore. The same situation is for me with smoking. I have few friends here, so I do not have to smoke in order to cater them.

Valuing relationship between friends; Smoking cigarettes for improving relationship with friends; Having less friends in the US; Being able to do his own things without impact from friends (personality?); Wishing to be a hero; Being impacted by the media; Regarding the hero in the movie as cool; Caring about the flavor of the cigarettes; Comparing his processes of drinking wine and smoking cigarettes; Giving up trying to drink wine and smoke cigarettes due to the unpleasant taste (personality: not being able to stick to his own opinion or his dream); Lacking the necessity of smoking after immigration

Acknowledging the benefit of quitting smoking (breathe better, rarely cough, function of lungs is better)

Lacking environment to smoke; Acknowledging the impact of environment on his smoking behavior; Believing personal smoking behavior can be impacted by environment and friends’ smoking behavior

Fearing to be viewed as a different person by friends; Changing personal behavior to look the same as his friends

Fang: What impact do you think it has on your life after you changed your smoking behavior?

Andrew: My feeling... I feel much better when I breathe. Sometimes I coughed before, but I rarely cough now. I feel the function of my lungs is much better.

Fang: Take a moment to think of some of the things that most affected your smoking habits. What has affected it the most? For example, after you came here, why you decreased your smoking amount? What are the things impacting your smoking behavior? Please tell me about that.

Andrew: After I came here, I had few friends here. There was no environment to smoke. It (My smoking behavior) has a relationship with the environment. When I was in Taiwan, a lot of friends smoked. I think that the environment where you live and your friends’ smoking behaviors really can impact you.

Fang: Mainly impacted by friends, right?

Andrew: Yes. If you stayed with them, you wish to be looked the same as them. You do not want them to think you to be a different person.

Fang: What about other things except for the impact from your friends?

Andrew: I do not think there are other things that impacted my smoking behavior. The main reason that I smoked was catering them.

Having no other reasons impacted his smoking behavior; Regarding catering friends as the main reason of his smoking

Not refusing that smoking helps relax stress (“do not disagree”); Explaining his understanding about the smoking and stress; Experiencing stresses and getting calm from smoking; Getting information on the consequences of smoking from TV (lungs changing black); Identifying nicotine as the source causing damage; Focusing on the long run benefit; Valuing health than relaxing stress

Getting cigarettes from his friends; Expressing confusion on the picture (a skeleton) of the package; Not knowing the purpose of the scary picture on the package; Not paying attention to the pictures on the package

Explaining his understanding about the scary picture (stands for death); Acknowledging the function of the scary picture on the package; Having knowledge about the consequences of smoking (causes cancers, impact lungs, leads to death); Being sensitive to the scary pictures

Fang: Some people think smoking can help them relax from stress, but some others think it is not good for health, how about your opinion on smoking?

Andrew: I do not disagree that (smoking can help people relax from stress). When you are stressed, your heart rate will be faster. But when you smoke, smoking can calm your emotion. I had this feeling. But the problem is that, in the TV programs, they show some of the smokers’ lungs changing black. Of course, it is caused by the nicotine, the chemical ingredient damaging the lungs. So, from psychological feeling, although smoking can relax stress, from the long run, I see the lungs changing like that. By considering the health, health is more important compared with relaxing stress.

Fang: Some people told me that the packages of the cigarettes in Taiwan often printed some scary pictures on them. What are you feeling about that?

Andrew: Sometimes my friends gave me the cigarettes. I do not know why the package of the cigarettes had a picture of a skeleton on it. But I only saw that once. I had no special experience at other times. I did not pay special attention to that.

Fang: Did the scary picture on the package of the cigarettes have any impact on you?

Andrew: Actually speaking, I think the skeleton stands for the death. In fact, it impacted me more or less. Given the truth that smoking causes cancers and impacts lungs in the long run, which leads to an early death, they are always related to each other. As for the picture of the skeleton on the package, it is sensitive for me more or less.

Fang: Can you give me an example to show that you think smoking benefit/harm you more than harm/benefit you?

Getting more harm than benefit from smoking; Linking smoking with his cough; Having cough for a long time; Being sensitive with his cough and lung cancer; Getting information on the consequences of smoking and smoking cessation from doctors

Getting information from Taiwanese doctors

Getting information during the physical health examination

Explaining the ingredients of the cigarettes (nicotine and tar); Having knowledge about the mechanism of cough; Considering cough as the direct consequence of smoking; Looking into long time effect (He is talking like an expert. Is he showing his knowledge?)

Getting information from media (internet, TV)

Andrew: I think smoking harms me more than benefits me. Because smoking can cause a cough. If it lasts for a long time...Cough, even for that, I had to see doctors before. I asked the doctor whether I had the problem of lung cancer. The doctor said if you kept on smoking, it was possible (for you to get lung cancer), but if you had not smoked a lot and quit as early as possible, it was not a big problem.

Fang: Did you see this doctor when you in Taiwan or here in the United States?

Andrew: In Taiwan. Before I came here, I went to see the doctor and the doctor asked me.

Fang: So, it was during the physical health examination. During the examination, the doctor asked you, right?

Andrew: Right.

Fang: How much do you know about the consequence of smoking?

Andrew: Smoking... it mainly includes nicotine and tar, the chemical ingredients causing the change of the lungs. They make the lungs secrete some discharges. The problem is that you smoke the chemical ingredients, the body has to discharge them. It causes a cough. In the long run, the function of the lungs will decrease when you smoke more. It will cause the irreversible damage to your lungs.

Fang: Where did you get the information regarding smoking consequence?

Andrew: Because... I care about the information in the media. Also, I often surf the internet. A lot of time, on the TV, they advocate the problem of quitting smoking.

Smoking offered cigarettes; Not smoking actively; Regarding quitting smoking as easy; Smoking less after coming to the US; Linking the degree of craving for the cigarettes with the difficulty of quitting smoking; Pointing his purpose of smoking (pleasuring his friends); Having a symptom of addiction; Having less relapse; Having a faster smoking cessation process; Acknowledging smoking is a habit; comparing smoking cigarettes with chewing gum

Pointing out e-cigarettes do not include nicotine; Identifying nicotine as the reason causing cancer; Disclosing information on e-cigarettes he got from media; Believing e-cigarettes are harmful; Agreeing to avoid e-cigarettes; Comparing the smell of e-cigarettes with cigarettes; Regrading the smell of e-cigarettes as not heavy and pleasant; Prioritizing the

Fang: For some people quitting is hard and for others it is easy. Some people begin smoking again soon after quitting and others do not. Some people quit many times or continue to smoke on and off again. Please tell me about your experience with quitting smoking. What has that been like for you?

Andrew: Because... Firstly, when I was in Taiwan, I smoked when they offered me the cigarettes. I did not smoke actively when they did not give me the cigarettes. So, it is not difficult for me (to quit smoking). Especially after I came to the US, I smoked less. For some people, they have smoked for a long time, and they have a craving for the cigarettes, so it is hard for them to quit. But for me, I smoked when they offered me the cigarettes, mainly for pleasuring them. In this situation... when I quit for a short period of time, I had the desire to smoke. But I did not have the relapse as frequently as the long-time smokers. So, I can quit faster. For example, some people like to chew gum. If they have a long history of chewing gum, they have the habit of chewing gum. But if they do not chew gum for a while, they will not have the desire.

Fang: Some people tried going to see a doctor, using the smoking cessation health service (e.g., smokers’ helpline), and smoking cessation assistant methods (e.g., e-cigarettes) to quit smoking. How was that in your case?

Andrew: Electronic cigarettes, of course, it did not have nicotine, the chemical ingredients causing cancers which are included in the cigarettes. But later, medical reports disclosed that although electronic cigarettes have less harm than traditional cigarettes, they still have some ingredients which are harmful to people. According to the medical reports, if we can avoid smoking electronic cigarettes, we should. But I did not refuse that the smell of the electronic cigarettes is not as heavy as that of cigarettes. Some of the electronic cigarettes smell very pleasant. But it has some chemical ingredients, so we should avoid smoking it as much as possible.

harm on health over the smell of e-cigarettes

Acknowledging more people from his circle smoked in Taiwan; Family members not smoking; Not all friends smoking

Getting advice of quitting smoking from doctors; Pointing the benefit of quitting smoking; Emphasizing personal awareness and personal will as a key point of quitting smoking (want); Addressing quitting smoking is a personal decision; Emphasizing the role of loved one in smoking cessation; Expressing the wish of change

Disclosing the doctor’s opinion about his smoking behavior (not smoking for a long time, not having the craving as others); Getting advice from doctors about quitting smoking

Comparing self-condition with the more addictive person; Being able to control personal desire to smoke; Having no desire to smoke without owing cigarettes

Fang: How many people from your circle (e.g., family members, friends, colleagues) smoke?

Andrew: In Taiwan, more people in my circle smoked. My parents, my older sister and younger sister do not smoke. Some of my friends smoke and some of they do not.

Fang: How much information have you got on smoking cessation from your doctors, nurses or community health workers?

Andrew: Usually they advise me to quit smoking and never smoke again. Although some of the people have lung diseases now, the condition of their lungs can improve a lot even at the beginning of the smoking cessation process. If they think smoking is harmful to their health, and they want to quit, they can quit. Other people cannot force them to do that. So, the key point is that---some people said that, which was impressive to me---quitting smoking for your loved one. Although I am single now and I do not have a girlfriend, I do not want to be in this condition for my whole life.

Fang: How much information have you got on smoking cessation from your doctors?

Andrew: Doctor... He said that I smoked not for a long time, and I did not have the craving as others, so he advised me to avoid smoking as much as possible.

Fang: So, your doctor had asked you and advised you to quit?

Andrew: Yes. But my condition is better than the people who smoke one package per day. It is hard for them to quit smoking. For me, I only smoked when my friends gave me the cigarettes. I did not want to touch the cigarettes if I did not have cigarettes.

Fang: How much information have you got on smoking cessation from your family members? For example, when your family members saw you smoking, what did they say?

Family members not seeing his smoking; Smoking with friends; Never smoking at home or by himself; Smoking to cater his friends

Contrasting the environment of smoking in casino; Being in the casino for more than ten years (gambling? related to his personality?); Regarding the smoke-free policy as good; Describing the smoke from cigarettes in casino as heavy; Being sensitive to the smoke; Linking second-hand smoke with nicotine; Imaging the impact of nicotine on his lungs; Describing himself as sensitive and fear of mysophobia; Preferring sitting in the smoke-free area; Regarding smoke-free policy is quite good for non-smoking people

Acknowledging the impact of smoking on human’s health; Agreeing with the smoking prohibiting policy (He introduced the new word “smoking prohibiting policy”, which was different from “smoke-free policy”. It seems he does not quite understand the difference between “smoking prohibiting policy” and “smoke-free policy”)

Emphasizing his limited number of cigarette smoking; Clarifying his personal preference on smoking (not like)

++

+

Andrew: This situation is rare. Because I often smoked with friends. When I stayed at home... Even by myself, I did not want to smoke. I smoked just for the purpose of catering my friends. I never smoked at home or smoked when I was alone.

Fang: How much do you know about California’s smoke-free policy?

Andrew: In earlier times, long ago, the casino allowed people to smoke. But later, about ten years ago, they prohibited people to smoke in the casino. People who smoke should go to the smoking area. I think this is good. Because when I was in the casino in earlier days, people smoked there. I smelt a heavy smoke. Frankly speaking, I am very sensitive. When I smoked the cigarette smoke, it reminded me of the nicotine. I inhaled the second-hand smoke, and I thought about its impact on my lungs. I am a sensitive person and fear of mysophobia. In this environment, I would better choose to sit in the smoke-free area. After California prohibited smoking in the public area, I think it is quite good for the non-smoking people.

Fang: So, you think California's smoke-free policy is good for you, right?

Andrew: Of course. Smoking has a huge impact on people's health. I quite agree with the smoking prohibiting policy.

Fang: Is there any change in your smoking behavior due to the smoke-free policy?

Andrew: For me, even considering the relationship of my friends, I still did not smoke so much. Basically speaking, I am not the person who likes to smoke proactively.

Fang: What are the barriers for you to quit smoking?

Andrew: The only barrier was that when my friends gave me cigarettes, I felt sorry to refuse them. I had to smoke in that situation. I did not want them to think me as a different person. But when I am not in this environment, for example, I am here in the US, no people smoke in my circle. I have no burden.

Regarding friends offering cigarettes as a barrier for his smoking cessation; Feeling pressures from refusing accepting friends’ cigarettes; Preferring to be looked as the same with his friends; Being feared to be left out; Lacking people from his circle smoke in the US; Feeling relax away from peer pressure in the US

Mentioning stages of lung cancer; Regarding early detection of lung cancer as safer; Addressing the importance of media reports; Getting information about the consequences of smoking from Chinese TV; Regarding advocating on the TV as effective; Pointing the cons of journals as an information delivery way (not everyone can see); Pointing the advantages of TV as an information delivery way (dynamic pictures, strong feeling)

Nearly quitting smoking after immigration; Only smoking in Taiwan

Linking meeting friends with smoking; Emphasizing his purpose of smoking (catering his friends); Stopping smoking for a long time

Fang: OK. That’s it. Is there anything else you want me to know about your experience and perceptions of smoking behavior change?

Andrew: From my view, lung cancer has different stages, such as the early stage, middle stage, and late stage. The earlier you know that you have lung cancer, the safer you are. Secondly, I think if the journals and TV do not report, people will not pay special attention. Like what I mentioned just now, I saw the people had a hole in his trachea. I thought whether it was caused by smoking. I saw this on the TV. It was a Chinese TV. So, I think it is effective to advocate on the TV. Because there are a lot of different kinds of journals. Not everyone can see the information in the journals. But people can see the dynamic pictures on the TV. The feeling is much stronger.

Fang: How many cigarettes do you smoke currently?

Andrew: I nearly quit the smoking after I come here. I smoked only when I was in Taiwan.

Fang: So, do you mean that I can say you have quit smoking, right?

Andrew: Yes, you can say that. For a long time after I came here, I did not meet with friends and smoke. Actually, when I was in Taiwan, I only smoked to cater my friends. So, I have stopped smoking for a long time.

Fang: OK. That is the end of the interview. Thank you for your answers.
